# Minimal volume for a fluid challenge in postoperative patients

**DOI:** 10.1186/cc14269

**Published:** 2015-03-16

**Authors:** H Aya, A Rhodes, RM Grounds, M Cecconi

**Affiliations:** 1 St George's Healthcare NHS Trust, London, UK

## Introduction

An effective fluid challenge should increase the mean systemic filling pressure (Pmsf ) in order to increase the venous return. The objective of this study was to determine the minimum volume of intravenous fluid required to significantly increase the Pmsf.

## Methods

Patients following cardiac surgery were randomly allocated to receive 1, 2, 3 or 4 ml/kg (body weight) of crystalloid over 5 minutes using a 60 ml syringe. Pmsf was measured using the arterial pressure after stopping blood flow in the arm with a pneumatic tourniquet inflated for 1 minute. Cardiac output (CO) was also recorded at baseline and immediately after the fluid infusion. CO was measured with LiDCO or pulmonary artery catheter, and a positive response was considered an increase of 10% from baseline. From previous data, the least significant change for Pmsf was 15%. Medians were compared using the independent samples media test, and proportions were compared using a chi-square test. Statistical significance was considered when *P *< 0.05.

## Results

Fifty patients were included, 40.8% of them were responders. The proportion of responders increases with the increase of dose of fluids (Table 1). The regression equation was: change of Pmsf (%) = 4.4 (dose of fluids ml/kg, 95% CI 2.3 to 6.5) - 1.6 (95% CI 7.4 to 4.3, *R*^2^ = 0.28, *F*(1.47) = 17.8, *P *< 0.001). The predicted dose of fluids required to achieve a change in Pmsf of 15% is 3.7 ml/kg crystalloids.

**Table 1:** Change of Pmsf-arm and CO.

	1 ml/kg (*n *= 12)	2 ml/kg (*n *= 12)	3 ml/kg (*n *= 13)	4 ml/kg (*n *= 13)	*P*
ΔPmsf-arm (%)	0.0 (-4 to 9)	6.5 (3 to 21)	9.0 (8 to 16)	18 (9 to 21)	0.05
ΔCO (%)	3.9 (0.4 to 10)	6 (2.1 to 9.1)	9.9 (-1.6 to 14.3)	12.9 (2.6 to 23.5)	0.1
Responders (%)	25.0	18.2	46.2	69.2	0.04

## Conclusion

The minimum volume required to perform an effective fluid challenge is 4 ml/kg infused in 5 minutes. However, only 30% of the variation of change in Pmsf can be explained by the dose of i.v. fluid given. The proportion of responders increases with the volume of fluids.

